# Downregulation of RIPK4 Expression Inhibits Epithelial-Mesenchymal Transition in Ovarian Cancer through IL-6

**DOI:** 10.1155/2021/8875450

**Published:** 2021-03-26

**Authors:** Huan Yi, Yan-zhao Su, Rong Lin, Xiang-qin Zheng, Diling Pan, Dan-mei Lin, Xiang Gao, Rong Zhang

**Affiliations:** ^1^The Third School of Clinical Medicine, Southern Medical University, Guangzhou 510500, China; ^2^Department of Obstetrics and Gynaecology, Fengxian Hospital, Southern Medical University, Shanghai 201499, China; ^3^Department of Gynaecologic Oncology, Fujian Provincial Maternity and Children's Hospital, Affiliated Hospital of Fujian Medical University, Fuzhou 350001, China; ^4^Key Laboratory of Maternity and Children's Major Diseases of Fujian Province, Fuzhou 350001, China; ^5^Fuzhou Maternity and Child Health Care Hospital, Fuzhou 350001, China

## Abstract

RIPK4 has been implicated in multiple cancer types, but its role in ovarian cancer (OC) has not been clearly elucidated. Our data from Gene Expression Profiling Interactive Analysis, RT-PCR, and immunohistochemical analysis showed that RIPK4 was expressed at higher levels in OC tissues and cells than in normal ovarian tissues and cells. Increased RIPK4 expression in OC markedly correlated with a worse overall survival than lower RIPK4 expression levels (hazard rate (HR) 1.5 (1.45–1.87); *P* = 0.001). In functional experiments, RIPK4 downregulation significantly inhibited metastatic behaviours in OC cells. Subsequently, based on data from 593 OC patients in the TCGA database, gene set enrichment analysis revealed that RIPK4 was involved in epithelial-mesenchymal transition (EMT) in OC. At the molecular level, silencing RIPK4 significantly downregulated vimentin, N-cadherin, and Twist expression but induced an increase in the protein level of E-cadherin and inhibited the IL-6 and STAT3 levels. Moreover, IL-6 levels were significantly decreased in RIPK4-silenced OC cells (*P* < 0.05). The addition of IL-6 to OC cells rescued the suppressive effect of RIPK4 knockdown on EMT. Thus, our data illustrate that downregulation of RIPK4 expression can restrain EMT in OC by inhibiting IL-6. This finding may provide a novel diagnostic and therapeutic target for improving the poor prognoses of OC patients.

## 1. Introduction

Ovarian cancer (OC) is the most fatal and third most frequent type of gynaecological malignancy worldwide [1]. Approximately 21,750 new OC cases and 13,940 OC deaths are projected to occur in the United States in 2020 [2]. The poor outcome of OC is largely driven by the high rate of intraperitoneal dissemination and extraperitoneal metastasis in newly diagnosed cases [3]. Epithelial-mesenchymal transition (EMT) is an initial and primary cellular step in malignant progression that is characterized by downregulated epithelial biomarker expression and upregulated mesenchymal biomarker expression [4–6]. However, the crucial transcriptional regulators that activate a variety of signal cascades to promote epithelial cell transformation into a mesenchymal phenotype in OC initiation and progression remain unknown. Thus, there is a pivotal and urgent need to elucidate the regulatory molecular mechanisms of OC progression, especially EMT.

A growing number of studies have revealed that the NF-kappa B (NF-*κ*B) and JAK/STAT signalling pathways are active in cancer-related EMT [7–10]. Another key pathway associated with EMT is the interleukin-6 (IL-6)/phosphorylated signal transducer and activator of transcription 3 (STAT3) axis [11]. IL-6 is an inflammatory factor widely reported to be involved in several pathological networks in cancer, and is also recognized as an activator of the STAT3 signalling pathway [11, 12]. This pathway has been identified to be associated with invasive abilities, such as angiogenesis, chemotherapy resistance, and cell cycle progression [13].

Receptor interacting protein serine/threonine kinase 4 (RIPK4), which is aberrantly expressed in multiple cancer types, is a key member of the group of receptor interacting proteins (RIPs) [14]. The RIPK4 protein has been observed to activate NF-*κ*B and is essential for keratinocyte differentiation [15, 16]. In addition, its related oncogenic signalling pathways include the NF-*κ*B, Wnt/*β*-catenin, and RAF1/MEK/ERK pathways [17, 18]. Previous studies have revealed that RIPK4 is overexpressed and promotes malignant progression in bladder urothelial, nasopharyngeal, cervical, colorectal, and pancreatic cancers [18–23]. However, the role of RIPK4 in OC is not clear and whether it can alleviate EMT through the IL-6/STAT axis is poorly understood.

Herein, we presented data showing the high level of RIPK4 in OC tissues and cells, which was found to be associated with a short overall survival (OS) time in patients with OC. Moreover, to further explore the RIPK4-related pathways involved in OC, gene set enrichment analysis (GSEA) was performed. The results revealed that downregulation of RIPK4 expression was able to hinder the EMT capacity of OC through IL-6, which might provide more specific evidence for the treatment of OC.

## 2. Materials and Methods

### 2.1. Cell Culture

Cancer cell lines (SKOV3, Coci, CAOV3, A2780, SW626, HEY, and OVCAR3) and the normal ovarian cell line IOSE80 were obtained from the Shanghai Cancer Institute. All cells were cultured in different culture media (SKOV3 in 5A (Gibco, New York, USA); Coci, CAOV3, A2780, SW626, and HEY in DMEM (HyClone Laboratories Inc., Logan, UT, USA); and IOSE80 in RPMI 1640 medium (HyClone Laboratories Inc., Logan, UT, USA)) supplemented with 10% (volume/volume) foetal bovine serum (FBS) (Gibco, New York, USA). OVCAR3 cells were cultured in DMEM (HyClone Laboratories Inc., Logan, UT, USA) supplemented with 20% (volume/volume) FBS. All culture media were supplemented with 1% double antibiotics (100 *μ*g/ml streptomycin and 100 U/ml penicillin). The cells were all cultured in a 37°C humidified incubator supplemented with a 5% CO_2_ atmosphere. Recombinant human IL-6 protein (Proteintech, Wuhan, China) was added to the OC cell culture medium after culturing.

### 2.2. Antibodies

The antibodies used in the study were purchased from ImmunoWay (Newark, DE, USA): mouse monoclonal anti-N-cadherin, mouse monoclonal anti-E-cadherin, mouse monoclonal anti-vimentin, rabbit monoclonal anti-STAT3, rabbit monoclonal anti-phospho-STAT3, and rabbit monoclonal anti-GAPDH. A mouse monoclonal anti-RIPK4 antibody was purchased from Santa Cruz Biotechnology (California, USA), and a rabbit anti-mouse IgG HRP-conjugated antibody was purchased from Abcam, Inc. (Massachusetts, USA). All antibodies were diluted to the highest concentration listed in the manufacturer's instructions.

### 2.3. Bioinformatic Analysis

The Gene Expression Profiling Interactive Analysis (GEPIA) website was used to show the RNA sequencing expression and survival analyses of OC based on thousands of samples from the TCGA and GTEx projects [24]. Kaplan-Meier plotter was used to assess the impact of RIPK4 on OC survival. GEPIA also provided the log rank *P* value and hazard ratio with 95% confidence intervals on each plot.

### 2.4. Knockdown of RIPK4 Expression in OC Cells

RIPK4 has two isoforms: RIPK4-201 (ENST00000332512.7) and RIPK4-202 (ENST00000352483.3). The length of RIPK4-201 was 832, and this isoform has been chosen as the canonical sequence. Different from the canonical sequence, 278-325 were missing in RIPK4-202. A lentivirus for downregulating the expression of both the RIPK4-201 and RIPK4-202 isoforms was purchased from Shanghai GeneChem Company, Ltd. (Shanghai, China). The interfering RNA (RNAi) sequences were as follows: si-1, 5′-TTTCACGTCCAGAT CATGAGC-3′; si-2, 5′-AAATCAGAAATCTTGACGTGG-3′; si-3, 5′-AAC AGGTCAATGACATCGGCG-3′; and si-control, 5′-TTCTCCGAACGTGTCAC GT-3′. The RNAi and control sequences were inserted into a plasmid vector (hU6-MCS-CBh-gcGFP-IRES-puromycin). The plasmids were transfected into cells with Lipofectamine 3000 (Invitrogen, Thermo Fisher Scientific, California, USA) following the instructions of the manufacturer.

### 2.5. Quantitative Real-Time PCR

Total RNA was extracted from cell lines by using TRIzol (Invitrogen, Thermo Fisher Scientific, California, USA). Reverse transcription was performed (Promega, Madison Wisconsin, USA) following the manufacturer's protocols. Subsequently, a 7300 Real-Time PCR Machine (Thermo Fisher Scientific, California, USA) with a SYBR Green PCR Master Mix (Takara, Osaka, Japan) was utilized, according to the manufacturer's instructions. The primer sequences for RIPK4 were as follows: forward, 5′-CATGA CCTCAGACGGCTAC-3′ and reverse, 5′-CCTTCTCCTCGACAAGCAG-3′. Beta-actin was used as an internal reference, and relative gene expression was analysed by using the 2^−ΔΔCt^ method.

### 2.6. Database and Gene Set Enrichment Analysis (GSEA)

Firstly, a gene expression matrix document containing 593 cases of OC patients were obtained from the TCGA dataset (https://www.cancer.gov/about-nci/organization/ccg/research/structural-genomics/tcga). Secondly, the gene expression matrix was divided into two groups according to the median expression of RIPK4 (high-expression group and low-expression group of RIPK4). Lastly, GSEA (http://www.broad.mit.edu/gsea/) was performed to explore the potential mechanisms underlying the effect of RIPK4. The gene set included *P* values, and a false discovery rate (FDR) less than 0.05 was regarded as remarkably enriched, and permutations were set 1,000 times for further analysis.

### 2.7. Patient Information and Clinical Specimens

Clinical information and specimens (Table 1) were retrieved from the Fengxian Hospital, Southern Medical University and Fujian Provincial Maternity and Children's Hospital, from 2004 to 2018. Since serous ovarian carcinoma is the most common pathological type of OC, we included 124 cases of serous ovarian carcinoma and 30 ovarian serous cystadenoma as controls. Paraffin-embedded specimens from patients who underwent surgery were confirmed by a specialized gynaecopathologist.

### 2.8. Immunohistochemical (IHC) Analysis

Tissues were sliced into 4 *μ*M sections for IHC staining. Antibody dilutions were 1 : 100 for the RIPK4 mouse polyclonal antibody (Novus, Missouri, USA) and 1 : 1,000 for the rabbit anti-mouse IgG HRP antibody (Abcam, Inc., Massachusetts, USA). The sections were incubated in xylene and a series of gradient concentrations of ethanol for dehydration and transparency following the manufacturer's recommendations. Endogenous peroxidase activity was blocked in 0.3% hydrogen peroxide for 10 minutes at 37°C, followed by washing with phosphate-buffered saline (PBS) for 3 minutes. After heating in 10 mmol/l citrate solution (pH 6.0) for 2 minutes to retrieve antigenic epitopes, slides were incubated with primary antibodies against RIPK4 at 4°C overnight. Then, the slides were washed with PBS three times, and the slides were incubated with secondary antibody for 1 h at room temperature. The tissue sections were visualized using the Enhanced HRP-DAB Kit (Tiangen Biotech, Beijing, China) by a Zeiss microscope. The sections were counterstained with hematoxylin after immunostaining, dehydrated with graded ethanol, cleared by xylene, and mounted with neutral balsam. Two pathologists who were blinded to the clinical information evaluated the immunoreactive scores (0~5% positive scored “0,” 6~25% positive scored “1,” 26~50% positive scored “2,” 51~75% positive scored “3,” and more than 75% positive scored “4”) and intensity (weak scored “1,” medium scored “medium” and strong scored “3”) independently. Finally, scores of 3~7 were defined as high expression, and scores of 0~2 were defined as low expression (a high-power field ×40 times with an area of 0.16 mm^2^).

### 2.9. Western Blotting

Cells were lysed with 1x IP lysis buffer (Solarbio, Shanghai, China) supplemented with Protease Inhibitor Cocktail (Solarbio, Shanghai, China). The total protein concentration was determined by using the BCA Protein Assay Kit (Beyotime, Shanghai, China). Then, cell lysates were combined with 5x SDS loading buffer (Solarbio, Shanghai, China) and boiled for 10 minutes, after which 20 *μ*g protein was loaded in each lane of a gel. After electrophoresis, the proteins were transferred onto nitrocellulose membranes and blocked in 5% BSA supplemented with 0.1% Tween. The membranes with proteins were incubated with mouse monoclonal antibodies against RIPK4 (ImmunoWay, Texas, USA), E-cadherin, N-cadherin, and vimentin (Cell Signalling Technology, Danvers, USA) and rabbit monoclonal antibodies against anti-phospho-STAT3 and GAPDH (ImmunoWay, Newark, DE, USA) diluted 1 : 1000 at 4°C overnight. After washing with PBS three times, an anti-rabbit HRP-conjugated antibody (goat anti-rabbit IgG, ImmunoWay) was then added and incubated for 1 h at room temperature. The FluorChem R System (ProteinSimple, California, USA) was used for imaging. Finally, the signals were quantified by using ImageJ.

### 2.10. Wound Healing Assay

For this assay, 1 × 10^6^ cells/well were plated in 6-well plates and cultured until approximately 95% confluent the next day. Wounds were made in the cell layer using sterile 1 ml pipette tips, and then the cells were washed twice with PBS. After washing, 2 ml FBS-free medium was added to the plate, and the scratch width was measured at 0, 24, 36, and 48 hours by using an inverted fluorescence microscope (Zeiss, Oberkochen, Germany). ImageJ software was used to quantitate the average wound width to calculate the decrease in scratch width (*μ*m) at each time point.

### 2.11. Transwell Invasion Assay

Chambers (8 *μ*m, Corning, New York, USA) were precoated with Matrigel (BD, NJ, USA). After treatment, 1 × 10^5^ OC cells in FBS-free medium were plated in the upper chamber of a 24-well plate, and 700 *μ*l medium containing 10% FBS was added to the basolateral chamber. After 48 hours of incubation, cotton swabs were used to remove the noninvaded cells, and the invaded cells were fixed with 4% paraformaldehyde (Solarbio, Shanghai, China) for 30 minutes and then stained with 0.1% crystal violet (Solarbio, Shanghai, China) in methanol for another 30 min. The stained chambers were then dried at room temperature overnight, and the invaded cells in the lower surface of the chambers were imaged in five random fields under an inverted fluorescence microscope (Zeiss, Oberkochen, Germany).

### 2.12. Subcutaneous Xenografts in Mice

Subcutaneous tumour xenografts were generated by implanting a total of 1 × 10^6^ SKOV3 cells (suspended in 200 *μ*l PBS) under the right flank skin of immunodeficient Balb/c mice. The mice were 5~6 weeks old at the start of experiments. The development of xenotransplantated tumour was evaluated, and palpable tumour volume = *π*/6 × length × width × height was measured every week. Mice were sacrificed after 8 weeks of treatment, and their tumour xenografts were moved, photographed, and fixed.

### 2.13. Enzyme-Linked Immunosorbent Assay (ELISA)

IL-6 levels in cell culture medium were measured by ELISA by following the protocol of the ELISA Max Deluxe Sets (BioLegend, USA).

### 2.14. Statistical Analysis

Functional results were analysed by Student's *t*-test or one-way ANOVA followed by the Bonferroni post hoc test. All experiments were performed in triplicate at a minimum. GraphPad Prism 7.0 was used for all statistical analyses, and *P* < 0.05 was considered significant.

### 2.15. Ethics Statement

This study was performed following the ethical norms contained in the Declaration of Helsinki. Patient consent and approval from the institutional ethics committee were acquired.

## 3. Results

### 3.1. A High Level of RIPK4 Expression Was Detected in OC

**Figure 1:**
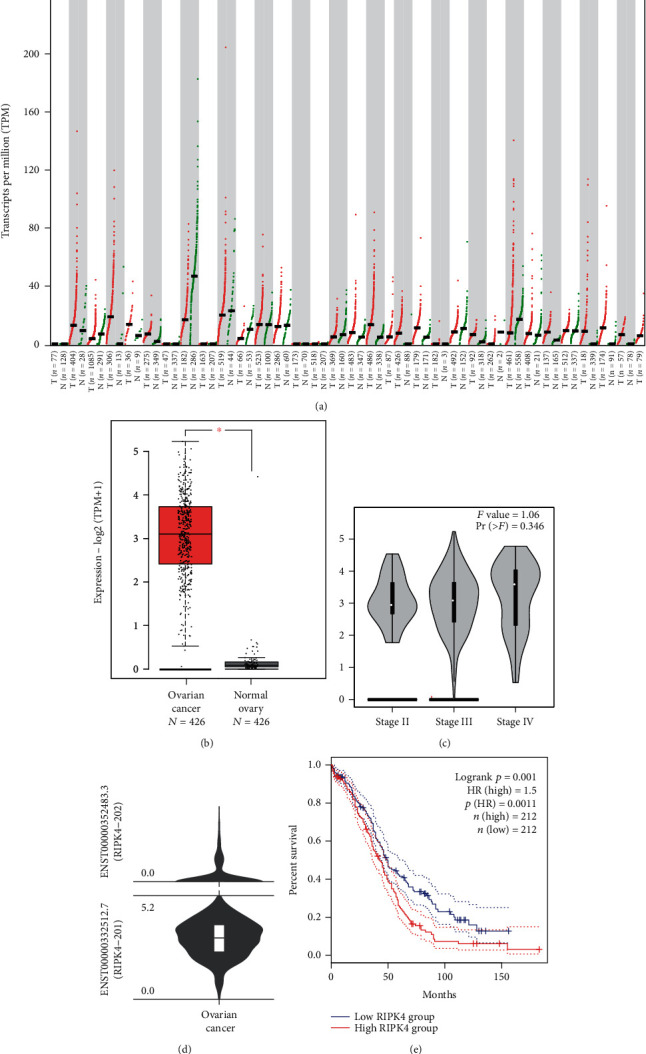
The expression and prognostic information of RIPK4 in OC, as analysed by GEPIA. (a) Large-scale expression profiling of RIPK4 in different types of carcinoma. (b) RIPK4 mRNA expression in OC patients (*N* = 426) was significantly higher than that in normal people (*N* = 426) (^∗^*P* < 0.05). The red column contains the OC tissues, and the black column contains the normal tissues. (c) To further identify whether RIPK4 expression is related to OC stage, GEPIA was used, which uncovered no significant difference among stages II-IV. (d) Violin plots indicate that the expression level of the RIPK4-201 (ENST00000332512.7) isoform of RIPK4 is significantly higher than that of the RIPK4-202 (ENST00000352483.3) isoform in OC. (e) The Kaplan-Meier plotter demonstrates that the high levels of RIPK4 in OC are associated with a significantly worse survival rate than low levels of RIPK4 expression (*P* = 0.0011).

First, the aberrant mRNA expression of RIPK4 in various cancer types compared with those of the corresponding control groups is depicted in Figure 1(a). Our previous research showed that OC is a highly heterogeneous carcinoma and has numerous molecular abnormalities [25]. Therefore, we further investigated the mRNA expression and prognostic role of RIPK4 in OC. The results showed that the mRNA expression of RIPK4 was significantly upregulated in OC tissues (Figure 1(b)) but was not related to the malignant stage of OC (Figure 1(c)). Moreover, the RIPK4-201 (ENST00000332512.7) transcript was significantly related to OC (Figure 1(d)). Furthermore, the Kaplan-Meier plotter showed that a high level of RIPK4 expression (HR 1.5 (1.45–1.87), *P* = 0.001) was associated with a worse overall survival than a low level of expression in OC patients (Figure 1(e)).

**Figure 2:**
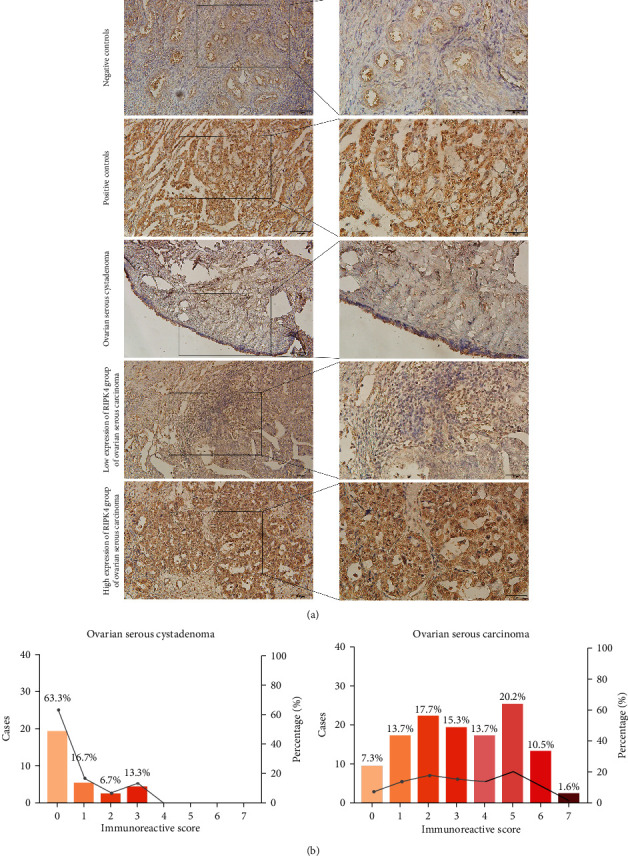
The expression of RIPK4 in protein level is available for all 154 cases. (a) IHC analysis shows that RIPK4 in cellular plasma is significantly higher in ovarian serous carcinoma than in ovarian serous cystadenoma tissues (*P* < 0.001). Besides, we present the isotype antibody staining in normal ovary as negative controls and ovarian serous carcinoma as positive controls. (b) RIPK4 immunoreactive scores for individual patients are presented.

To further analyse the RIPK4 expression in protein level in clinical samples, we revealed a significantly elevated expression of RIPK4 in HOGSC compared to normal ovarian tissues by IHC analysis. The patient clinical data including 124 (80.5%) patients with ovarian serous carcinoma and 30 (19.5%) patients with ovarian serous cystadenoma are summarized in Table 1. The relationship between RIPK4 expression and histological type and age of enrolled benign and malignant serous ovarian tumours, as well as histological grade, lymph node metastasis, and clinical stage of serous ovarian cancer was further studied (Table 2). The results verified that the higher expression of RIPK4 was related to the type of ovarian histology, and the expression in serous carcinoma tissues was significantly higher than that in ovarian serous cystadenoma (*P* < 0.001). In serous ovarian cancer, the expression of RIPK4 in high-grade cancer tissues was obviously higher than that in low-grade cancer tissues, and lymph node metastasis and late clinical stage were also significantly correlated with high RIPK4 immunological reactivity (*P* = 0.003, respectively). High immunological reactivity of ovarian serous carcinoma samples was 58.1% (124 patients: 48 low expression, 76 high expression), while that of ovarian serous cystadenoma tissues was 13.3% (patients: 26 low expression, 4 high expression) (*P* < 0.001, Figure 2).

### 3.2. Silencing RIPK4 Restrained the Migratory and Invasive Abilities of OC Cells

**Figure 3:**
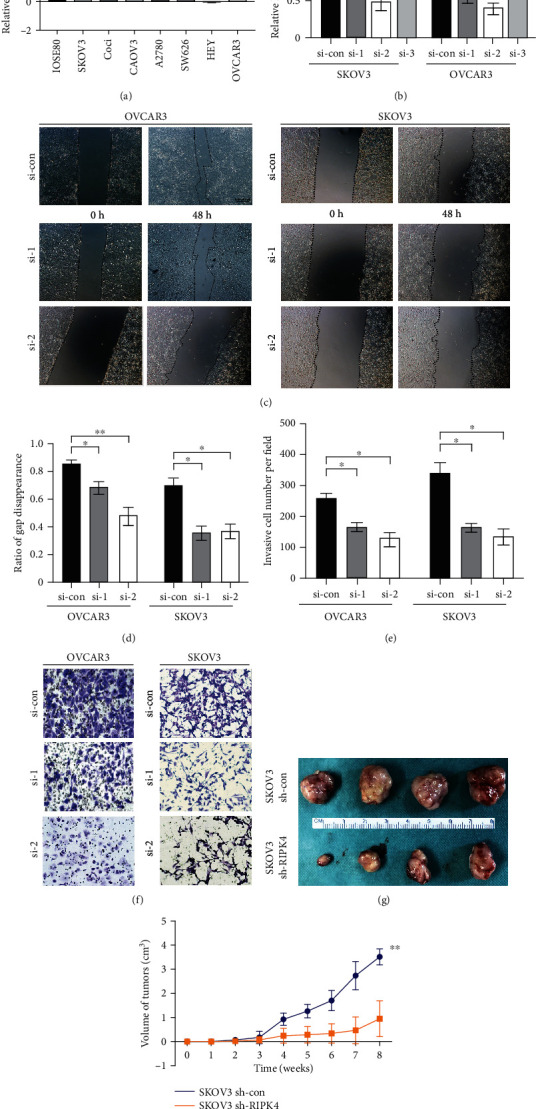
Silencing RIPK4 restrains OC cell migration and invasion. (a) RT-PCR was performed to assess the expression of RIPK4 in OC and normal ovarian cells. (b) RIPK4 silencing was carried out by using siRNA in SKOV3 and OVCAR3 cells. Silencing RIPK4 in OC cells significantly decreased their migratory ability, as observed in a wound healing assay (c and d), and their invasive ability, as observed in a Transwell assay (e and f). Images of subcutaneous xenografts are shown, *n* = 8 (g), and the tumour volumes of the SKOV3 subcutaneous xenografts in the RIPK4-silenced group and control group are shown (h). The data are shown as the mean ± SD, and statistical significance is indicated as follows: ^∗∗^ represents *P* < 0.01 and ^∗^ represents *P* < 0.05 (unpaired *t*-test).

The mRNA expression levels of RIPK4 in OC cell lines (SKOV3, SW626, CAOV3, A2780, OVCAR3, Coci1, and HEY) and a normal ovarian epithelial cell line (IOSE80) were detected by RT-PCR (Figure 3(a)). The results showed that in both OC tissues and OC cell lines, RIPK4 expression was notably upregulated. To explore the function of RIPK4 in OC, RNAi-RIPK4 was used to inhibit RIPK4 expression in OC cell lines (Figure 3(b)). Through wound healing (Figures 3(c) and 3(d)) and Transwell assays (Figures 3(e) and 3(f)), the mobility-related capacities of SKOV3 and OVCAR3 cells were shown to be obviously decreased by RIPK4 silencing compared with si-control treatment.

### 3.3. Silencing RIPK4 Inhibited the Growth of Subcutaneous Xenografts

EMT is an important step in OC invasion and distal colonization. The final result of EMT is metastasis and proliferation. Subcutaneous xenografts can show the RIPK4-related EMT effect on the malignant proliferation of OC. To determine whether silencing RIPK4 inhibits the growth of SKOV3 cell xenografts in immunodeficient mice, we established a total of four subcutaneous xenografts to compose the SKOV3-sh-RIPK4-silenced group, while another 4 xenografts were established with SKOV3 vector cells in immunodeficient mice as controls. All mice were sacrificed at the 8th week after injection. As shown in Figures 3(g) and 3(h), the OC xenografts in the SKOV3-sh-RIPK4-silenced group were dramatically smaller than those in the control groups (*P* < 0.01), which verified that RIPK4 could promote the growth of OC in vivo.

### 3.4. Identification of RIPK4-Related Signalling Pathways

**Figure 4:**
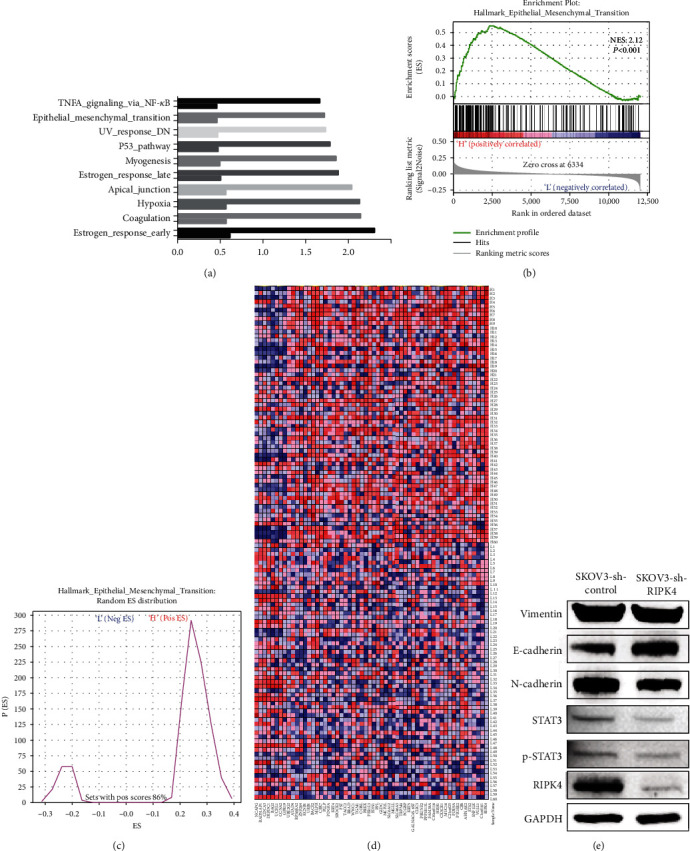
GSEA results. (a) GSEA identified the top ten pathways in the high RIPK4 expression group based on the TCGA dataset including data from 593 OC patients. (b and c) GSEA showed that the high RIPK4 expression group was associated with EMT. (d) Gene profiling of hallmarks is shown in the heatmaps generated by comparing the high RIPK4 expression group and the low RIPK4 expression group. (e) Western blot assay results demonstrated that the levels of proteins promoting the EMT pathway were decreased in SKOV3-sh-RIPK4 cell lines.

Functional assays demonstrated that RIPK4-silenced OC cells exhibited suppressed tumour growth, migration, and invasion. To explore the functional pathway involving RIPK4 in OC, GSEA was utilized to assess signalling pathways involved in OC in low and high RIPK4 expression groups. GSEA identified significant differences (NOM *P* value < 0.05, FDR < 0.05) in the enrichment of hallmarks. The results revealed that NF-*κ*B, EMT, the p53 pathway, myogenesis, apical junctions, and hypoxia were the top differentially enriched pathways in the high RIPK4 expression phenotype that were closely related to the oncogenesis and progression of OC (Figure 4(a)). EMT has been identified as a classic and influential cellular process in OC progression, regardless of whether tumours are in an early or late tumour stage. Analysis of RIPK4 mRNA expression also showed no significant difference in response to OC stage, so we focused on EMT for biological and pathway studies. For EMT in the GSEA results, the enrichment score (ES) was 0.55, and the normalized enrichment score (NES) was 2.12 (Figures 4(b) and 4(c)). In addition, a heat map illustrated that the hallmarks of EMT related to the high RIPK4 expression phenotype were largely upregulated, as shown in Figure 4(d).

### 3.5. RIPK4 Silencing Suppressed EMT in OC Cells

To further confirm the correlation between RIPK4 and EMT in OC cells, WB and RT-PCR assays were performed with SKOV3 and OVCAR3 cells to detect the expression of vimentin, E-cadherin, and N-cadherin, which are regarded as the main mesenchymal markers or epithelial markers during EMT. The results in Figure 4(e) show that in RIPK4-silenced OC cells, vimentin and N-cadherin expression was significantly downregulated, while E-cadherin expression was correspondingly upregulated. These data indicated that knockdown of RIPK4 expression suppressed EMT in OC cells.

### 3.6. RIPK4 Promoted IL-6/STAT3-Mediated EMT in OC Progression

**Figure 5:**
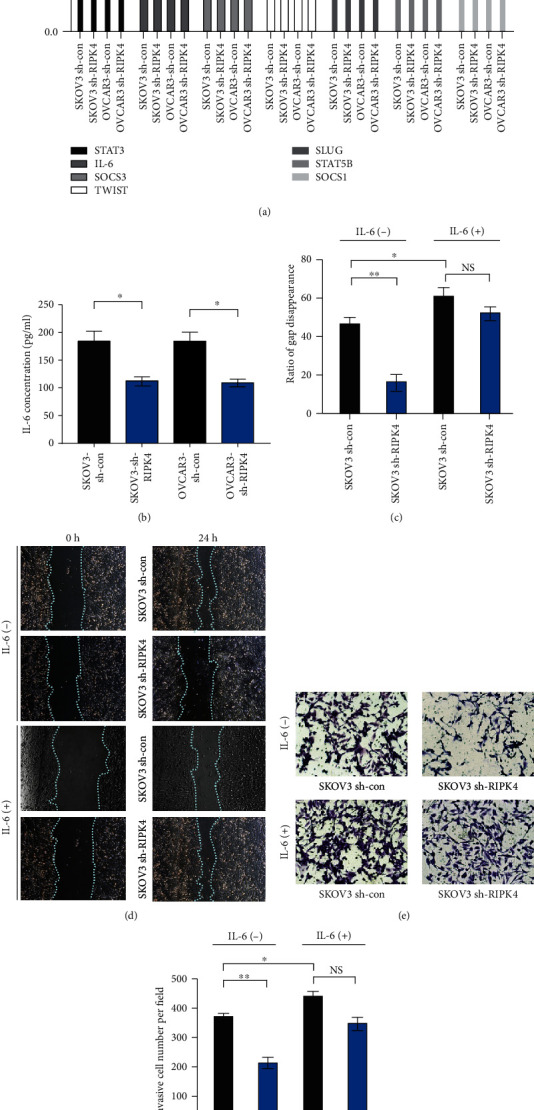
RIPK4 affects the IL-6/STAT3 axis in OC, and IL-6 promotes SKOV3 migration and rescues the mobility of RIPK4-silenced cells. (a) RT-PCR results show that the levels of IL-6 target genes and EMT biomarkers are downregulated in RIPK4-silenced OC cell lines compared with control cell lines. The results are shown as the mean ± SD (*n* = 6 per group). (b) ELISA analysis shows that the IL-6 levels in the supernatants of RIPK4-silenced OC cell cultures were significantly lower than those in control culture supernatants (*P* = 0.036). (c and d) The effects of adding IL-6 (50 *μ*g/ml) to SKOV3-sh-RIPK4-silenced and control OC cells were detected with wound healing assays. IL-6 significantly promoted migration by SKOV3-sh-RIPK4-control cells (*P* = 0.018). (e and f) Additionally, IL-6 rescued the migratory ability of SKOV3-sh-RIPK4-silenced cells (*P* = 0.051). The ratio of gap disappearance and the invasive cell number is shown. ^∗∗^*P* < 0.01 vs. control (unpaired *t*-test). ^∗^*P* < 0.05 vs. control (unpaired *t*-test).

We further explored the RIPK4-related mechanisms involved in EMT in OC. Based on previous studies that revealed that RIPK4 can activate the NF-*κ*B pathway, that STAT3 is essential for NF-*κ*B activation, and that activating the IL-6/STAT3 pathway results in EMT [7–12], we aimed to determine whether RIPK4 induces EMT through IL-6 in OC cells. We abolished RIPK4 expression by silencing with shRNA sequences and then performed WB and RT-PCR assays. In the RIPK4-silenced OC cells, STAT3 and p-STAT3 levels were significantly downregulated, as determined by WB assays (Figure 4(e)). In addition, the levels of STAT3, IL-6, and the IL-6 target genes SOSC1 and SOSC3 were dramatically decreased by RT-PCR assays. Furthermore, the levels of STAT5B and mesenchymal biomarkers (Slug and Twist) were also significantly decreased in the RIPK4-silenced OC cells (Figure 5(a)). To quantify the decreased level of IL-6 in RIPK4-silenced OC cells, we tested the concentration of IL-6 in the culture medium by ELISA. As shown in Figure 5(b), the concentration of IL-6 in the medium is significantly downregulated in the medium of SKOV3-sh-RIPK4 cells (*P* = 0.036).

### 3.7. IL-6 Administration Rescued the Migration and Invasion of RIPK4-Silenced OC Cells

**Figure 6:**
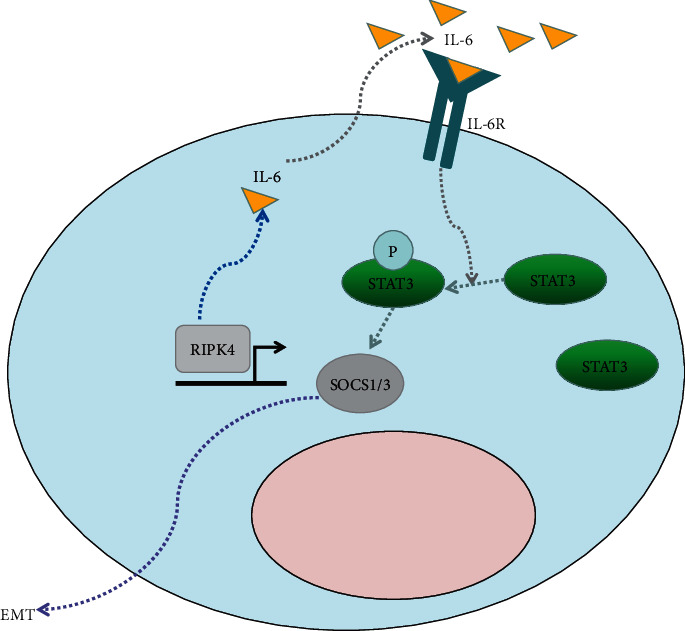
Functional model of the IL-6 involved in restraining EMT in OC progression after downregulation of RIPK4 expression. RIPK4-silenced OC cells hypoactivated IL-6 signalling. Less STAT3 was phosphorylated, and the expression of SOCS1/3 was reduced. Consequently, the RIPK4-based IL-6 network is a determinant of EMT in OC.

To determine the potential role of IL-6 in RIPK4-induced EMT, we added IL-6 to RIPK4-silenced OC cells. As demonstrated in Figure 5(a), IL-6 significantly promoted the migration of SKOV3-RIPK4-control OC cells. In addition, IL-6 rescued the migration (Figures 5(c) and 5(d)) and invasion (Figures 5(e) and 5(f)) of SKOV3-RIPK4-silenced OC cells. These data verified that RIPK4 induced EMT via IL-6 in OC cells (Figure 6).

## 4. Discussion

Approximately 70% of ovarian cancer cases are found to involve metastasis to the abdominal cavity at initial diagnosis. Most patients die of malnutrition and intestinal obstruction secondary to the abdominal metastatic niche [26]. EMT is an early and vital cellular step in ovarian epithelial cancer oncogenesis and progression [27–29]. To date, the key regulatory factors and underlying tumourigenic and metastatic functions of EMT remain poorly understood. It was reported that the activation of NF-*κ*B and the IL-6/STAT3 pathway can promote EMT in a variety of cancers and that blocking NF-*κ*B reduces metastasis in breast cancer. In addition, previous studies have demonstrated that RIPK4 can activate the NF-*κ*B pathway and promote malignant behaviours in epithelial carcinomas. However, whether RIPK4 induces EMT through the IL-6/STAT3 pathway still needs to be explored in malignant epithelial cancers, particularly in OC. Our results demonstrated that RIPK4 expression was significantly upregulated in OC cells and tissues, and a high level of expression was closely related to poor clinical survival. Furthermore, we revealed by GSEA that RIPK4 induced EMT in OC. For the first time, we demonstrated that RIPK4 promoted EMT in OC by targeting IL-6, thus providing a novel molecular mechanism for early diagnosis and a potential target for the treatment of abdominal metastasis in OC.

RIPK4, located on chromosome 21q22.3, is a serine/threonine protein kinase with C-terminal region 11 ankyrin repeats and N-terminal RIP-like kinase domains [21]. Currently, the role of RIPK4 in cancer tumourigenesis and the developmental role of RIPK4 remain obscure, and its role in OC is far from clear. In the present study, the increased expression of RIPK4 was shown in both OC tissues and OC cell lines, which significantly predicted a poor prognosis in OC. Notably, a high level of RIPK4 expression was also detected in three common gynaecological cancers and in testicular germ cell tumours. The existing literature has demonstrated the differential expression and prognostic role of RIPK4 in malignant tissues. For instance, similar results for the expression and prognostic value of RIPK4 were reported in pancreatic cancer, cervical cancer, and colorectal cancers [18, 19, 22]. In contrast, decreased expression of RIPK4 was found in hepatocellular carcinoma and tongue squamous cell cancer [23, 30]. However, these studies did not provide an extensive view of RIPK4 in malignant tumours. Overall, our TCGA data-based study initially demonstrates the potential oncogenic role of RIPK4 in three common gynaecological cancers. In addition, bioinformatic analysis showed no significant difference in RIPK4 expression among OC stages, which might suggest early involvement of RIPK4 in OC oncogenesis and development.

EMT has been identified as an early and important step in epithelial carcinoma. Activation of NF-*κ*B has been found to be a key promoter of EMT in liver cancer [31], colon cancer [32], gastric cancer [33], and various other cancers [34]. In the current study, functional assays illustrated that silencing RIPK4 in OC cell lines significantly restricted migratory and invasive properties. Previous studies have indicated that RIPK4 can activate the NF-*κ*B pathway and RAF1/MEK/ERK signalling in cancer oncogenesis and development [21]. De Oliveira et al. revealed that a decreased ability to undergo EMT to repair skeletal muscle undergoing repair was related to downregulation of RIPK4 expression [35]. However, these authors did not investigate the top potentially enriched pathways between RIPK4 and malignancy based on a large-scale number of OC patients. In contrast to all previous work, we have been the first to provide new insights into the critical role of RIPK4 as an oncogene involved in the p53 pathway, myogenesis, apical junctions, hypoxia, and in particular, EMT by GSEA analysis of data from 593 OC patients in a TCGA dataset. In the current study, RIPK4 silencing was accompanied by downregulation of the expression of epithelial biomarkers (vimentin, Slug, N-cadherin, and Twist) and upregulation of the expression of a mesenchymal biomarker (E-cadherin).

Activation of the IL-6/STAT3 pathway can block antitumour immunity in cancers. We initially demonstrated that RIPK4 silencing induced the downregulation of IL-6 and STAT3 expression, as well as that of the IL-6 targets SOSC1 and SOSC3 [36]. IL-6 is secreted by various cancer cell types and cancer-associated fibroblasts, causing chronic inflammation in tumours, which then binds IL-6R to form the IL-6/IL-6R complex [36]. This complex elevates phosphorylated STAT3 (p-STAT3) expression and activates nuclear translocation-induced effector genes, contributing to inflammation, immune reactions, and cancer oncogenesis and development [37]. In addition, IL-6/STAT3 signalling is essential for NF-*κ*B activation. However, previous studies did not address whether RIPK4 activates IL-6 to induce NF-*κ*B activation in malignancies. Notably, we revealed that silencing RIPK4 inhibited IL-6 and SOCS1/3. In addition, adding IL-6 was able to rescue the migratory and invasive abilities of RIPK4-silenced OC cells, which confirmed the regulatory role of RIPK4 in the IL-6/STAT3 pathway.

## 5. Conclusions

It is worth noting that our study had certain limitations. First, the identification of RIPK4 was based on the TCGA database, but we did not perform an in vivo study to further confirm that RIPK4 can induce EMT in OC. Second, the potential links between RIPK4 and hormones, such as oestrogen and androgen, were not investigated in our study.

Overall, this study reveals that the RIPK4-based IL-6 network is a determinant of EMT in OC and provides a novel diagnostic, therapeutic, and prognostic biomarker for OC.

## Figures and Tables

**Table 1:** Basic clinical information of patients with serous ovarian cancer and benign ovarian serous cystadenoma.

	Ovarian serous carcinoma (*n* = 124)	Ovarian serous cystadenoma (*n* = 30)
Age		
Min, max	18-82	18-68
Median	55	44
Histological grade of ovarian serous carcinoma		
High	74	
Low	50	
Clinical stage		
I	35	
II	12	
III	68	
IV	9	

**Table 2:** Clinicopathological features and expression of RIPK4 in enrolled patients.

Characteristic	Total cases, *n* = 154 (%)	RIPK4 expression		*χ* ^2^ value	*P* value
		Low-expression cases (%)	High-expression cases (%)		
Histological type					
Ovarian serous carcinoma	124 (80.5%)	48 (38.7%)	76 (61.3%)	22.256	<0.001
Ovarian serous cystadenoma	30 (19.5%)	26 (86.7%)	4 (13.3%)		
Age					
≤50	59 (38.3%)	30 (50.8%)	29 (49.2%)	0.002	0.969
>50	95 (61.7%)	48 (50.5%)	47 (49.5%)		
Histological grade of ovarian serous carcinoma					
High	74 (59.7%)	21 (28.4%)	53 (71.6%)	8.256	0.004
Low	50 (40.3%)	27 (54.0%)	23 (46.0%)		
LN metastasis					
Yes	31 (25.0%)	5 (16.1%)	26 (83.9%)	8.882	0.003
No	93 (75.0%)	43 (46.2%)	50 (53.8%)		
Clinical stage					
I	35 (28.2%)	22 (62.9%)	13 (37.1%)	14.013	0.003
II	12 (9.7%)	5 (41.7%)	7 (58.3%)		
III	68 (54.8%)	20 (29.4%)	48 (70.6%)		
IV	9 (7.3%)	1 (11.1%)	8 (88.9%)		
Clinical stage					
I	35 (28.2%)	22 (62.9%)	13 (37.1%)	12.891	0.002
II	12 (9.7%)	5 (41.7%)	7 (58.3%)		
III-IV	77 (62.1%)	21 (27.3%)	56 (72.7%)		
Clinical stage					
I-II	47 (37.9%)	27 (57.4%)	20 (42.6%)	11.200	0.001
III-IV	77 (62.1%)	21 (27.3%)	56 (72.7%)		

## Data Availability

Data is available through the authors themselves.
